# Brain Metastasis in a Young Patient with Uterine Carcinosarcoma

**DOI:** 10.7759/cureus.5010

**Published:** 2019-06-26

**Authors:** Vatsala Katiyar, Tiago Araujo, Muhammad Z Farooq, Ishaan Vohra, Shweta Gupta

**Affiliations:** 1 Internal Medicine, John H Stroger, Jr. Hospital of Cook County, Chicago, USA; 2 Hematology / Oncology, John H Stroger, Jr. Hospital of Cook County, Chicago, USA

**Keywords:** uterine carcinosarcoma, malignant mixed mullerian tumor, mmmt, cerebral metasatasis, cns metastasis, endometrial cancer

## Abstract

Uterine carcinosarcoma occurs almost exclusively in post-menopausal women and often carries high rates of disease recurrence and mortality. Central nervous system involvement is extremely rare with less than 10 reported cases in the literature. Here we present a young pre-menopausal patient with metastatic malignant mixed Mullerian tumor who developed cerebral metastasis while on systemic chemotherapy.

## Introduction

Uterine carcinosarcoma (UCS) is a high grade and rare uterine malignancy with overall incidence being less than 5% of all malignant uterine neoplasms [1]. Risk factors include exposure to radiation, tamoxifen use, black race, obesity, nulliparity and exogenous estrogen use [1-2]. This metaplastic tumor is known to metastasize extensively, however central nervous system involvement is an unusual occurrence. Management options for brain metastasis in UCS can be surgical resection, stereotactic radiosurgery (SRS) and whole brain radiation therapy (WBRT) with corticosteroid use depending on the number and site of lesions [3].

## Case presentation

A 41-year old Hispanic lady, gravida 0, with diabetes, hypertension, and a prolonged history of metrorrhagia presented to us with continuous heavy vaginal bleeding for three months. She also complained of cramping pain in lower abdomen, bloating, nausea, non-bloody and non-bilious vomiting and 15-pound weight loss during the same time period. Her family history was remarkable for pancreatic cancer in mother and maternal cousin diagnosed at 68 and 32 years, respectively; breast cancer in maternal aunt at age 70; another maternal cousin at age 50 years; and uterine cancer in maternal grandmother at an unknown age. She did not want to be tested for genetic mutations.

**Figure 1:**
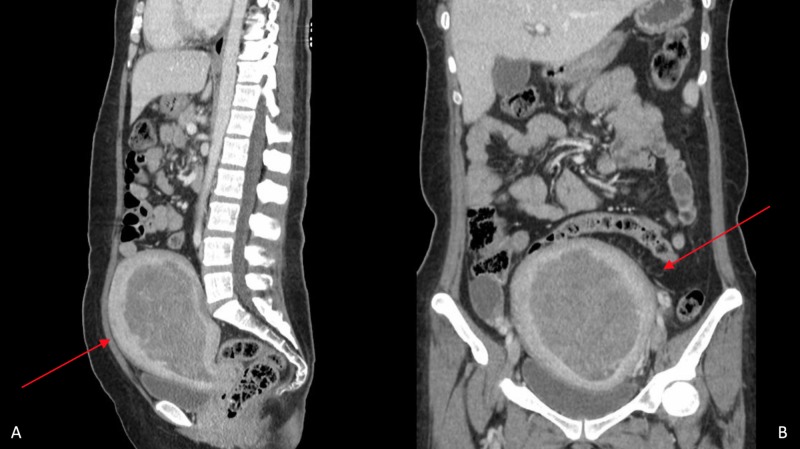
Sagittal and coronal views of the CT scan of the abdomen/pelvis with contrast Panes A (sagittal) and B (coronal) reveal diffuse distention and heterogeneous attenuation centered within the endometrium with diffuse enlargement (15 x 13.5 x13 cm) of the uterus (red arrows).

**Figure 2:**
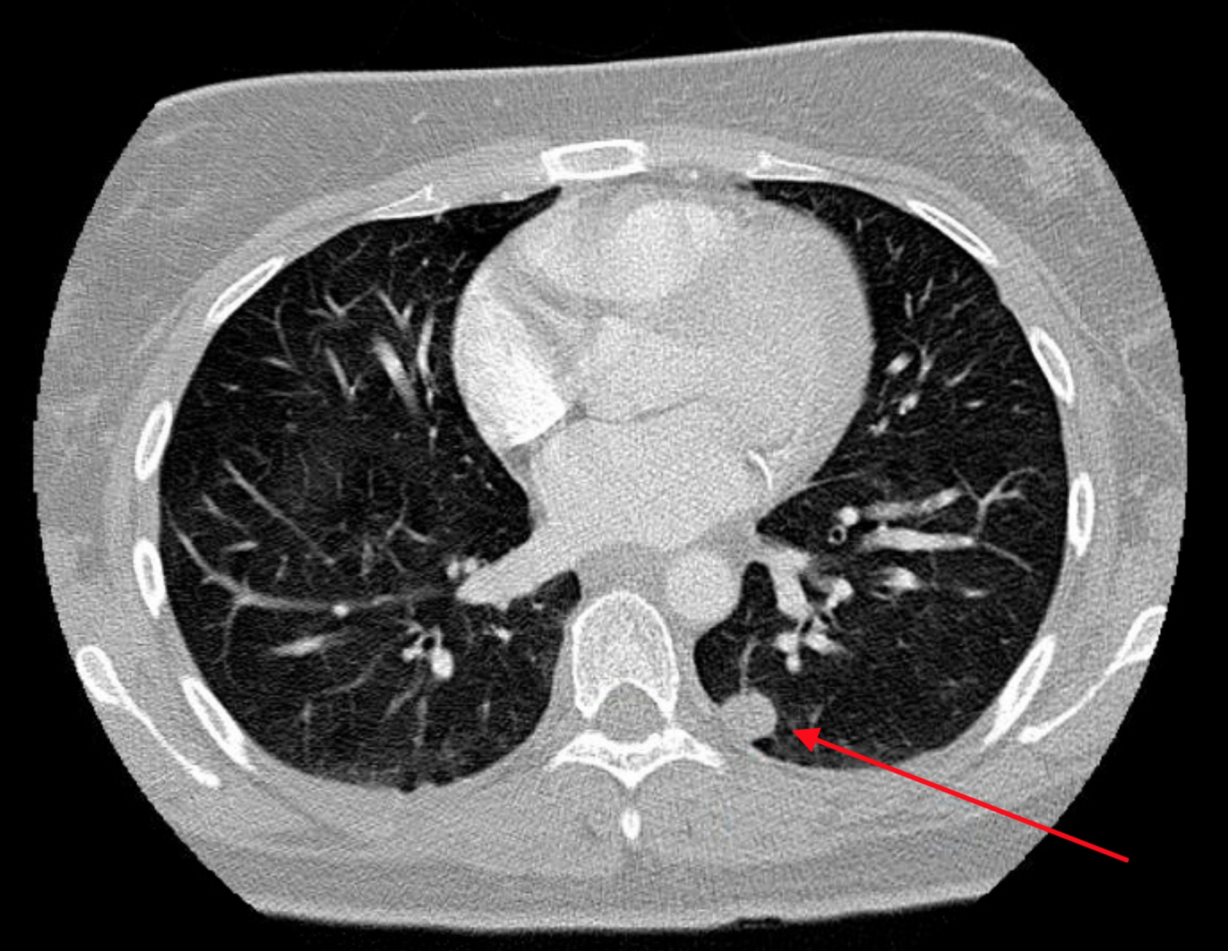
Axial view of computed tomography scan of the chest with contrast Red arrow points to largest metastatic lung nodule located in the left lower lobe and measuring approximately 1.1 cm in the long axis.

Physical examination was remarkable for an enlarged uterus approximately 18 weeks in size. There was no inguinal lymphadenopathy. The vaginal examination revealed a small amount of blood in the vaginal vault and no other palpable cervical, vaginal, or adnexal masses. Laboratory results were significant for hemoglobin of 7.2 g/dl (normal 11.7-14.9 g/dl), ferritin 19 ng/mL (11-306.80 ng/ml), carcinoma-antigen-125 (CA-125) of 61 U/ml (normal <35 U/ml), and carcinoembryonic antigen (CEA) of 3.16 ng/ml (normal <3.0 ng/ml). The CT scans of the chest, abdomen, and pelvis revealed diffuse enlargement of the uterus abutting the bladder (Figure 1) showing bilateral hydronephrosis, multiple external iliac and para-aortic lymph nodes and multiple bilateral pulmonary nodules (Figure 2), consistent with metastatic disease. The CT scan of the head was unremarkable. Pathology from uterine biopsy revealed high-grade malignant cells with sections showing atypical glandular cells admixed with spindled and chondroid cells, consistent with the diagnosis of uterine carcinosarcoma (malignant mixed Mullerian tumor).

Due to the large size of the primary tumor and metastatic nature of the disease, she was started on systemic chemotherapy with ifosfamide and paclitaxel. Her symptoms, including vaginal bleeding, improved with four cycles, but she still continued to have recurrent episodes of heavy bleed, requiring intermittent blood transfusions. The uterine size decreased on subsequent CT scans. However, the tumor was still deemed unresectable by the surgical oncologist as there was a risk of bladder injury during surgical intervention. Hence, two more cycles of chemotherapy were administered, which she tolerated well. 

**Figure 3:**
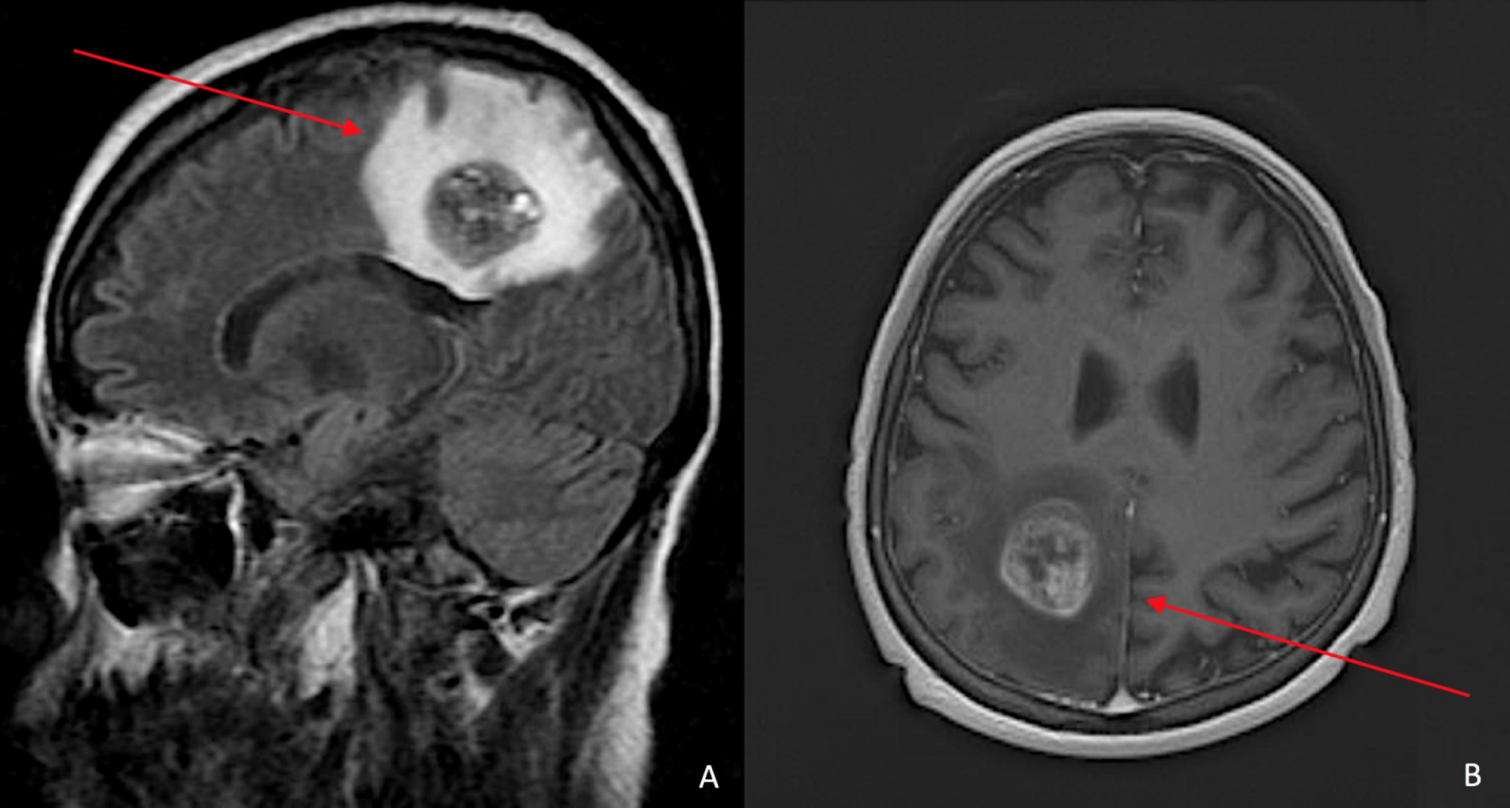
MRIs of sagittal (A) and axial (B) views of the brain Heterogeneous mass in the parasagittal right parietal lobe just above the posterior body of the corpus callosum (red arrows). It measures 3.0 x 2.7 x 2.5 cm and demonstrates peripheral and septated internal enhancement of IV contrast. There is a large surrounding perilesional vasogenic edema that causes mild mass effect and partial effacement of the posterior horn of the right lateral ventricle with slight inferior displacement of the posterior body of the corpus callosum.

Three weeks after receiving the last dose of ifosfamide and paclitaxel and just before re-staging the CT scan, she presented with new progressive left-sided arm and leg weakness. A CT-scan of the head was obtained and revealed a new metastatic lesion on the right posterior frontal lobe, 2.9 x 2.3 cm in size and with associated vasogenic edema. MRIs of the brain showed a 3.0 x 2.7 x 2.5 cm hemorrhagic and enhancing mass with large surrounding vasogenic edema in the parasagittal right parietal lobe (Figure 3). The CT scan of the chest and abdomen revealed a significant increase in size and number of metastatic lung lesions, with concomitant worsening of uterine mass, and multiple necrotic lymph nodes within the abdomen and pelvis. 

Dexamethasone was started, which led to some improvement of her weakness. She was deemed to be a non-surgical candidate by neurosurgeons due to her worsening systemic disease and functional decline. Palliative whole brain radiation therapy with 30 Gy in 10 fractions was started. However, her performance status declined rapidly, and she showed up inconsistently for radiation therapy. She eventually expired at home within a month of diagnosis of brain metastasis, and eight months after diagnosis of the primary disease.

## Discussion

Uterine carcinosarcoma (UCS), also known as malignant mixed Mullerian tumor (MMMT) is a rare but aggressive neoplasm with high rates of recurrence and distant metastases despite treatment [2]. Common sites of extrauterine spread include lymph nodes, ovaries, fallopian tubes, and omentum [4]. Similar to other uterine and gynecologic malignancies, brain metastases have rarely been described [5]. To our knowledge, eight other cases of MMMT with central nervous system (CNS) involvement have been reported [3]. Compared to those, our patient was the youngest to develop brain metastasis from MMMT at age 41.

MMMT has both epithelial and mesenchymal elements and was traditionally categorized as a sarcoma. However, a growing body of evidence suggested that the sarcomatous component is, in fact, derived from the epithelium, prompting reclassification as dedifferentiated endometrial carcinoma [3]. While the epithelial component of this metaplastic tumor has been most frequently identified at the metastatic foci, most cases of CNS involvement showed a predominant sarcomatous differentiation [2-3]. A brain biopsy was not pursued in our patient, given her overall declining status. Therefore, the exact histologic composition could not be established.

UCS/MMMT is considered primarily a disease of post-menopausal women with the median age at diagnosis being 70 years [6]. The median interval from presentation to CNS involvement and median survival, thereafter, have been reported to be 4.5 months and 3 months, respectively. Sarcomatous predomination has been linked to shorter survival [3]. Our patient was 41-year-old at diagnosis, brain metastasis occurred seven months after presentation, and she expired in the following month. Predictors of poor prognosis in this high-grade malignancy include tumor size, lymph node metastasis, adnexal spread, lymphovascular space involvement, depth of invasion of myometrial wall, as well as histologic cell type and grade [7]. 

Management of uterine carcinosarcoma is multimodal and requires coordination between surgeons, oncologists, and radiation therapists. Since it accounts for less than 5% of all uterine malignancies [1], treatment is largely extrapolated from trials done on patients with carcinomas and/or sarcomas [3]. Nonetheless, survival benefits have been demonstrated, with complete gross resection of the tumor even in advanced disease [8]. When CNS metastases are present, survival over 24 months has been described in two of the eight cases reported in the literature, and both had surgical resection of the metastatic lesion (one in left frontal and the other in intramedullary C2 level) [3]. Hence, the initial treatment of this disease should ideally be cytoreductive surgery involving hysterectomy, bilateral salpingo-oophorectomy, retroperitoneal lymphadenectomy, and omental biopsy, and if CNS metastasis is present, it should be considered for resection (especially if isolated), provided the patient is a surgical candidate. 

Due to the large size of the primary tumor, risk of bladder compromise with surgery, and the presence of metastatic disease, our patient first underwent chemotherapy with ifosfamide and paclitaxel. Of all chemotherapy regimens studied for UCS, this combination has shown a clear survival benefit with improved median overall survival from 8.4 to 13.5 months (p = 0.03) when compared with ifosfamide alone, which is the agent with best response rate when used in isolation [9]. While radiation therapy by itself decreases the risk of pelvic recurrences and time to distant spread, it does not confer a survival benefit in early stages of the disease and should not be used alone in advanced stages due to its inability to control distant foci of metastases [10-11].

In patients with CNS involvement, therapy is individualized due to a lack of standard management guidelines. However, based on limited available data, resection followed by WBRT is indicated in cases of solitary brain metastases as it improves overall survival, performance status, and local control when compared to WBRT alone [12-13]. However, in case of multiple lesions or unresectable disease, palliative corticosteroids or radiation therapy is preferred [14]. Resection was not considered in our patient due to poor response to chemotherapy, worsening metastatic disease burden and declining functional status. 

## Conclusions

In conclusion, MMMT is a rare tumor most often seen in post-menopausal females. It is associated with high mortality and has poor response to treatment. It frequently metastasizes but central nervous system involvement is exceedingly rare. While debulking surgery and chemotherapy with ifosfamide and paclitaxel has shown a survival benefit, complete remission is rare. Targeted treatment strategies for UCS are being studied, but so far have not been successful. Further understanding the molecular and genetic nature of the disease will aid in the evolution of therapeutics.
